# Successful Combination Therapy of Optic Canal Decompression and Steroid Administration for Traumatic Optic Neuropathy in a 10-Year-Old Boy

**DOI:** 10.7759/cureus.70124

**Published:** 2024-09-24

**Authors:** Hiroshi Fukumasa, Yurie Yamaga, Ryo Miyaoka, Masashi Kobayashi, Kazutaka Nishiyama

**Affiliations:** 1 Department of Pediatrics, Kitakyushu City Yahata Hospital, Kitakyushu, JPN; 2 Department of Critical Care and Anesthesiology, National Center for Child Health and Development, Tokyo, JPN; 3 Department of Neurosurgery, Hospital of the University of the Occupational and Environmental Health, Fukuoka, JPN; 4 Department of Traumatology and Acute Critical Medicine, Osaka University Hospital, Osaka, JPN

**Keywords:** optic nerve decompression, systemic steroid therapy, trauma pediatric, traumatic optic neuropathy (ton), treatment

## Abstract

Traumatic optic neuropathy (TON) is a rare complication caused by head injury in children. TON treatment has employed conservative treatment, steroid administration, and surgical procedures; however, which treatment is preferable remains controversial. We herein present a case of a 10-year-old boy with a TON-complicated head injury after falling from a two-meter-high slide in a park. Initial head computed tomography (CT) revealed the right optic canal fracture, and the patient complained of right visual impairment. He was diagnosed with TON, and surgical right optic canal decompression was performed at six hours post-injury. On postoperative day 2, his right visual acuity (VA) was 20/200, and his right eye developed a relative afferent pupillary defect, prompting a high-dose prednisolone administration. On day 12 post-injury, his right VA improved to 20/30. This clinical course suggests that a combined approach of optic canal decompression and steroid therapy was effective in this case. Further investigation is needed to identify optimal treatments that contribute to favorable visual outcomes for TON management in children. However, in pediatric patients, aggressive treatment may be warranted to prevent permanent visual impairment, with decisions made based on individual background factors and neurological symptoms.

## Introduction

Traumatic optic neuropathy (TON) is a rare condition, occurring in 0.5-5% of head injuries [1]. Similar incidence rates have been reported in pediatric patients [2].

One of the causes of TON is an optic canal fracture. Typically, an indirect force from trauma to the lateral brow region leads to an optic canal fracture. This results in primary injury, including the rupture of optic nerve fibers, followed by secondary injuries such as microcirculatory disturbances, hemorrhage, and edema. These effects cause compression and strangulation of the optic nerve within the optic canal [3-6].

Given the pathophysiology of TON, the primary objective of treatment is to achieve decompression within the optic canal, a confined space, to prevent further compression of the optic nerve. Current decompression approaches include surgical optic canal decompression, steroid therapy, and a combination of both [5-14]. However, there have been reports of spontaneous recovery of visual acuity (VA) with conservative management alone in some cases, and the effectiveness of steroid therapy and surgical intervention for visual recovery remains a topic of ongoing debate [3,15,16]. Consequently, some studies have suggested that treatment strategies should be individualized based on the specific clinical circumstances of each patient [4,15-18].

Despite the rarity of TON and the lack of consensus on optimal treatment, TON can cause severe and permanent visual impairment. As permanent vision loss can significantly reduce the quality of life in children throughout their lives, early diagnosis and treatment of TON are critical. Although there have been reports questioning the efficacy of surgical intervention and steroid therapy, we herein present the case of a 10-year-old boy with TON, in whom an aggressive treatment approach combining early surgical intervention and steroid administration resulted in a favorable VA prognosis.

## Case presentation

A 10-year-old boy presented to our emergency department (ED) with blunt head trauma and a right lateral eyebrow abrasion following a fall from a two-meter-high slide in a park. On initial examination, he was alert, with a Glasgow Coma Scale score of 15 (E4V5M6). His vital signs on admission were as follows: heart rate, 108 beats/min; blood pressure, 129/74 mmHg; respiratory rate, 13 breaths/min; oxygen saturation on room air, 98%; and body temperature, 37.3°C. The patient exhibited no motor or sensory deficits in the extremities; however, his right pupil demonstrated slightly sluggish reactions to light. Hematology test results indicated that hemoglobin levels and platelet counts were almost within normal limits. However, aspartate aminotransferase, alanine aminotransferase, lactate dehydrogenase, and D-dimer levels were elevated (Table 1).

**Table 1:** Laboratory analyses RT-INR: prothrombin time-international normalized ratio; APTT: activated partial thromboplastin

Parameter	Value	Normal range
White cell count (cells/μL)	13,000	3,300-8,600
Hemoglobin (g/dL)	13.4	13.7-16.8
Platelet count (cells/μL)	349,000	158,000-348,000
Aspartate aminotransferase (U/L)	64	13-30
Alanine transferase (U/L)	50	10-42
Lactate dehydrogenase (U/L)	327	124-222
PT-INR	1.03	0.9-1.3
APTT (seconds)	23.8	24-32
Fibrinogen (mg/dL)	243	200-400
D-dimer (μg/ml)	10.45	≦1.0

**Figure 1:**
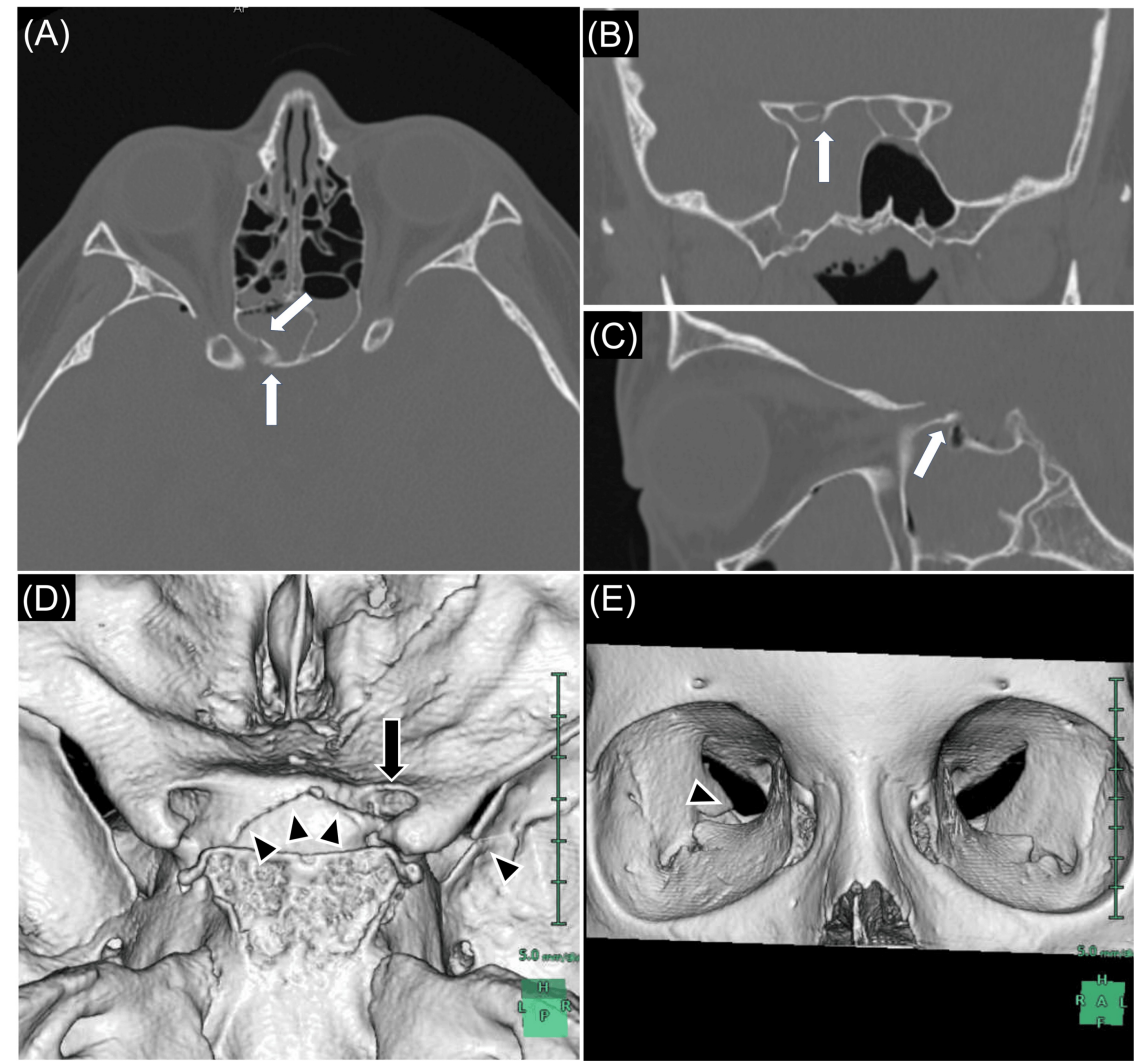
Bone window computed tomography (CT) at the initial examination (A): Axial view showing a right orbital wall fracture (white arrows) and pneumocephalus; (B): Coronal view showing that the fracture caused right optic canal narrowing (white arrow); (C): Sagittal view showing fractures at the right optic canal (white arrow) and pneumocephalus; (D) and (E): Three-dimensional CT (3D CT) revealing the fracture (black arrows) and the subsequent right optic canal narrowing (black arrowheads).

**Figure 2:**
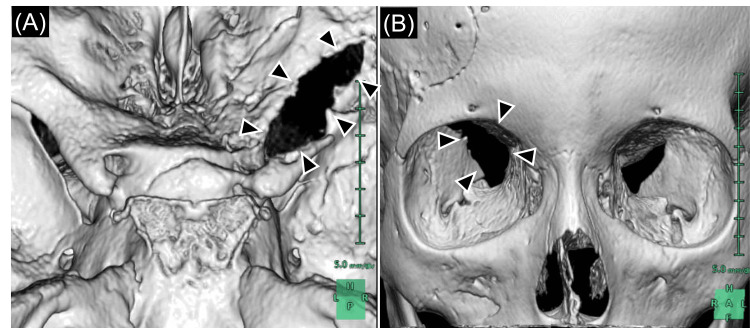
Three-dimensional computed tomography (3D CT) taken following optic canal decompression (A, B): 3D CT revealing the decompression of the right optic canal postoperatively (black arrowheads).

Head computed tomography (CT) revealed a fracture of the right optic canal and a narrowing of the right optic foramen compared to the left side (Figure 1). Upon re-examination, the right pupil continued to show sluggish responses to light, and the patient complained of blurred vision in his right eye compared to the left. A detailed vision examination by an ophthalmologist could not be performed because it was a Saturday. Based on his direct right optic canal fracture, the patient was diagnosed with TON and underwent surgical decompression of the right optic canal six hours post-injury (Figure 2). Preoperatively, VA in the right eye, initially at finger counting in the ED, had deteriorated to hand motion. The surgery was completed without any complications.

**Figure 3:**
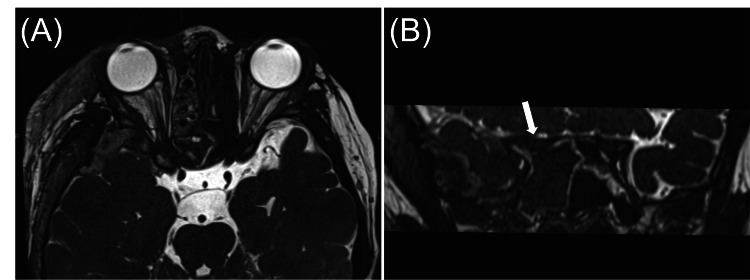
Constructive interference in steady state (CISS) magnetic resonance imaging (MRI) of the orbit on postoperative day 2 MRI (A: axial view; B: coronal view) showing no abnormalities in the right intra-orbital and optic nerve (white arrow).

On postoperative day 1, the patient reported some visual improvement compared to the immediate post-injury state. On postoperative day 2, an orbital magnetic resonance imaging (MRI) scan (Figure 3) showed no abnormalities in the right orbit or optic nerve. Despite this, the patient experienced significant vision loss in the right eye (VA: 20/200 (R) versus 20/16 (L)). Examination by an ophthalmologist revealed a relative afferent pupillary defect (RAPD) in the right eye. Consequently, high-dose prednisolone (25 mg/kg/day) was administered to prevent further optic nerve edema.

After three days of steroid treatment, the patient’s vision improved to 20/50 in the right eye. Steroids were gradually tapered over the following two weeks, and VA in the right eye improved to 20/30 by day 12 post-injury. The patient was discharged on day 16. No postoperative complications such as cerebrospinal fluid leak or meningitis were observed during hospitalization. At a nine-month outpatient follow-up, the patient’s VA remained at 20/30 in the right eye, which was an improvement from the post-injury state but still below his pre-injury VA of at least 20/20.

## Discussion

Head and facial trauma are common injuries in children presenting to the ED. However, TON associated with these injuries is rare, with an incidence reported as 0.5%-5% in adults [1]. The incidence in children is comparable, with a reported annual incidence of approximately one in 100,000 [2]. Despite its rarity, TON can cause severe and permanent visual impairment. As permanent vision loss can significantly reduce the quality of life in children throughout their lives, early diagnosis and treatment of TON are critical. In some cases, children with TON may also sustain brain injuries resulting in coma. In these instances, vision loss due to TON may be overlooked because the primary focus of post-injury treatment is the brain injury. Even in cases of head trauma without loss of consciousness, TON might be missed due to challenges specific to children, such as their inability to verbally express complaints of vision loss [7]. This issue is particularly relevant for infants and young children. In the case of our patient, despite the initial finding of a sluggish right pupillary light reflex, he did not report any visual abnormalities during the initial examination. It was only after head CT revealed an optic canal fracture that he first complained of vision abnormalities by himself during a follow-up medical interview, leading to a diagnosis of TON. For cases involving infants who cannot articulate vision complaints by themselves, suspecting TON based on the mechanism of injury (e.g., falls, motor vehicle accidents) and the injury site (e.g., external brow) and an aggressive search for optic nerve canal fractures using head CT following the initial examination is crucial [7]. Additionally, VA examinations, including RAPD, fundus examination, and visual evoked potentials, should be conducted by an ophthalmologist as early as possible.

Although several studies have investigated the efficacy of steroid therapy, surgical optic canal decompression, and conservative treatment, there is still no consensus on the optimal management of TON [3,4,16-19]. A Cochrane review found no evidence supporting optic canal decompression for TON, and decisions regarding surgical intervention should be made based on the potential benefits in each case [15]. Moreover, the indications for optic canal decompression have been debated, with factors such as (1) the presence of an optic canal fracture, (2) the timing of surgical intervention post-injury, and (3) VA at the time of injury being correlated with postoperative visual outcomes. Some studies have reported that surgical intervention can improve VA when an optic canal fracture is present [8]. Additionally, several studies suggest that early optic canal decompression is associated with a better VA prognosis [5,9]. This benefit of early intervention has also been reported in pediatric cases of TON, where early surgery has led to favorable visual outcomes [7,10,11]. In the present case, optic nerve canal decompression was performed within six hours post-injury, which may have contributed to the patient’s favorable VA recovery. It has also been noted that postoperative VA improvement rates are higher when the patient's preoperative VA is better than hand motion [12] or light perception [9]. In this case, the patient’s VA was better than hand motion in the preoperative evaluation, suggesting a good postoperative visual prognosis. No postoperative complications were observed in this patient. However, it is important to note that complications such as cerebrospinal fluid leakage and infection can occur postoperatively [3]. Therefore, the decision to proceed with surgery should be made cautiously. Nevertheless, in pediatric TON cases, early surgical intervention may be considered, particularly when an optic canal fracture is present and the initial post-injury VA is better than light perception.

Several studies have reported negative outcomes associated with steroid therapy for TON [17,19]. Additionally, some reviews have concluded that, due to the relatively high rate of spontaneous visual recovery in TON, steroids offer no significant benefit over conservative treatment [3,16]. Furthermore, a previous study indicated that steroid therapy may not only be ineffective but potentially harmful [8]. However, other studies have demonstrated the potential efficacy of steroids in the treatment of TON [13,14]. Gupta et al. noted that the optic nerve canal is smaller in the pediatric population, providing less space for the nerve to expand compared to adults [10]. As a result, children may be more susceptible to secondary damage from optic nerve compression, making steroids, which are believed to reduce optic nerve edema, potentially more beneficial for TON in pediatric cases than in adult cases. Therefore, the use of steroids in children with TON should not be entirely ruled out.

Rajiniganth et al. reported that the combination of optic canal decompression and steroid therapy resulted in improved VA outcomes in adult patients with TON [6]. In pediatric TON, a previous study recommended early intervention rather than waiting for spontaneous recovery, with combined surgical intervention and steroid therapy improving VA in approximately 80% of patients [10]. In this case, although MRI revealed no significant abnormalities in the optic nerve, the patient's VA did not show substantial improvement by postoperative day 2. To prevent further secondary damage due to optic nerve edema, steroids were administered after confirming the absence of postoperative infection and cerebrospinal fluid leakage. The steroid therapy was completed as planned without any complications, and the patient's VA improved compared to the early postoperative period. In this case, the combination of optic canal decompression and steroid therapy appeared to be effective.

Unfortunately, neither surgery, steroids, nor their combination has consistently proven effective for the treatment of TON. To date, the decision to perform optic canal decompression should be made on a case-by-case basis, taking into account the patient’s background and the extent of optic nerve damage. However, children with permanent visual impairment from TON may require more aggressive treatment than adults, as permanent visual impairment due to TON may significantly reduce the quality of life in children throughout their long lives during both childhood and adulthood. To borrow Valpo’s expression [20], if these treatments for TON are relatively safe and scientific evidence exists, then, ultimately, “what would I want if this happened to my son or daughter?” may be the driving force behind the treatment.

## Conclusions

We presented the case of a 10-year-old boy with TON who experienced a favorable treatment course and significant improvement in VA following early optic canal decompression combined with postoperative high-dose steroid therapy. However, high-quality evidence supporting any specific treatment for TON, including conservative approaches, remains lacking. Further research is needed to establish evidence-based guidelines for the optimal management of TON. In the meantime, aggressive treatment may be considered in pediatric TON cases, with careful consideration of the patient's background factors and neurological symptoms when determining indications for intervention.
